# Insight into the Extractive Metallurgy of Tin from Cassiterite

**DOI:** 10.3390/ma17133312

**Published:** 2024-07-04

**Authors:** Allen Yushark Fosu, Danièle Bartier, Frédéric Diot, Ndue Kanari

**Affiliations:** Université de Lorraine, CNRS, GeoRessources, F-54000 Nancy, France; daniele.bartier@univ-lorraine.fr (D.B.); frederic.diot@univ-lorraine.fr (F.D.)

**Keywords:** cassiterite, tin, extractive metallurgy, stannic oxide, stannous oxide, dry chlorination, alkaline leach, acid leach

## Abstract

This review details both the conventional and emerging methods of extracting tin from cassiterite. The emerging methods reviewed include sulphuric acid leaching of SnO, cooling crystallization of SnO, sulphide leaching, alkaline leaching, and dry chlorination. From these methods, the conventional approach (direct reduction smelting) stands out as the sole method that is suitable for industrial application, with none of the emerging ones being promising enough to be a contender. The thermodynamics involved in the hydrometallurgical extraction of tin from the mineral are also discussed. ${\Delta G}^{o}$ values calculated at 25 °C for the reduction–dissolution of SnO_2_ using reducing gases revealed feasibility only when carbon monoxide was used. An indication of the possible species produced during the hydrolysis of the oxide of the metal (SnO_2_ and SnO) as a function of pH (ranging from −2 to 14 and 0 to 14 for SnO_2_ and SnO, respectively) was noted and highlighted to link a Pourbaix diagram generated from literature data. This diagram suggests that the solubility of SnO_2_ in both strongly acidic and alkaline media is possible, but with a small dissolution window in each. The purification and recovery routes of the various processing techniques were then envisaged.

## 1. Introduction

Cassiterite (SnO_2_) is the most economically viable minerals for extracting tin among the over fifty minerals that host the metal [1,2,3,4]. Being the only oxide mineral, its theoretical tin content is 78.77%; however, in the ore form, the metal may show a value between 0.4 and 1.5% [1,5]. Considering primary sources, cassiterite finds itself as the main source of the metal before considering its sulphidic counterparts. For instance, cylindrite (Pb_3_Sn_4_FeSb_2_S_14_), stannite (Cu_2_FeSnS_4_), franckeite (Pb_5_Sn_3_Sb_2_S_14_), etc., are common tin-bearing minerals, but they are of less economic importance [1]. Tin-bearing ores may either be alluvial (placer) or hard-rock deposits, with those found in Indonesia, Thailand, and Malaysia being mostly alluvial, whilst Australia, China, and South America host the hard-rock deposits [3,6]. The alluvial type is simply beneficiated by gravity separation using spirals, shaking tables, and gravimetric concentrators (such as the Falcon and Knelson concentrators), with advantages due to its high density, coarse size, and easy liberation, but the hard-rock type requires complex beneficiation. Thus, the rock undergoes size reduction and screening (classification) following gravity separation and flotation to achieve a concentration of the mineral of about 60 to 70% [1,2,3,7,8]. Table 1 gives the composition of major elements (as oxides) of an Egyptian cassiterite concentrate containing 76 wt% of SnO_2_ [9].

Complex concentrates obtained after beneficiation that have polymetallic composition are often pretreated to remove undesirable components such as lead, arsenic, iron, sulphur, etc., either by roasting in an oxidizing environment, pressure leaching using HCl, or carbochlorination [2,10]. Pretreatment by roasting helps to oxidize sulphidic minerals and separate sulphur as SO_2_ through volatilization, whilst leaching solubilizes unwanted metallic materials [2,10]. By carbochlorination pretreatment, iron is specifically volatized as iron chloride when the concentrate is treated below 1000 °C. The downside of this approach is the volatilization of tin alongside ferric chloride. Both volatized products (chloride of tin and iron) can, however, be recovered, and the ferric chloride can be used as a subsequent chlorinating agent.

Apart from the primary sources discussed above, the metal is sourced secondarily from electronic equipment and tailings, contributing 30 to 50% of global production [2]. Tin is one of the oldest metals known to man, having been around since the early days of the Bronze Age, where it was found to exhibit different characteristics when mixed with copper to form bronze specifically for the fabrication of cutting tools, sculptures, and weapons [2,5,11]. The metal’s high corrosion resistance, malleability, and low melting point makes it useful for tinplating (coating reactive metals with tin to prevent corrosion), food packaging applications, and soldering (when alloyed with Pb) [5]. The metal has a global resource estimation of around 4.9 million tonnes [6]. It is considered one of the rare and critical base metals due to its low concentration (~2 ppm in the earth crust) compared to others like copper, lead, and zinc, which means it ranks 49th in terms of mineral abundance [5,12,13]. In 2014, its annual global production and consumption was about 300,000 to 400,000 tons, with China and Indonesia producing the largest portion (totalling around 70%) [10,14].

Studies on the extractive metallurgy of tin are scarce, which is partly attributable to the confidentiality of smelters’ operations, low global production, and the difficulty in developing technical expertise [13]. Feed, for tin smelters, generally consists of concentrates obtained after beneficiating the ore to increase the mineral composition (usually more than 60%) [6,7]. Reverberatory furnaces are often used among tin smelters, but the electric and blast furnace types are also used to some extent.

The reductive smelting of cassiterite (conventional process), which is currently the method of choice for the industrial extraction of tin from cassiterite, faces two major challenges: (1) the difficulty in separating iron oxide, Fe_2_O_3_, without affecting the recovery of the metal, and (2) process inefficiencies resulting from equipment design [2,13]. The former arises from the reduction of concomitant iron oxide in the concentrate to metallic iron (Fe) and/or wustite (FeO) and their entrainment to the crude tin as Fe or to the slag as FeO. The wustite and tin (II) oxide in the slag have similar chemistry regarding free energy of formation, atomic radius, charge, etc. They, therefore, have similar behaviour, specifically regarding the degree of dissolution in slag and reduction, which makes their separation difficult [13]. Dissolved Fe in the crude metal is separated as a mushy dross by scooping off; the entire dross cannot be discarded, but a portion (which may contain Fe) is recirculated since it usually contains some tin. This makes the total separation of the two metals by this approach a challenge. The latter challenge results from improper heat transfer through the charge in the smelter due to its design. There is, therefore, an excessively high energy requirement that needs to be met to achieve appreciable recovery alongside the loss of a significant amount of heat. This results in exorbitantly high operating costs. Several kinds of furnaces (the laboratory, rotary, and electric types) with several equipment arrangements have been investigated as ways to reduce or prevent the aforementioned inefficiencies [15]. Top-submerged lance technology, as talked about by Kandalam and co. [16], is typically used and has been integrated and/or coupled with the conventional reverberatory furnace as a way of mitigating the aforementioned challenges. Elsewhere, the passage of a suitable gas through the molten slag to enhance the thorough and efficient mixing of the charge was suggested in [13].

Irrespective of the measures taken to curb these challenges, the process produces slag with high iron and appreciable tin content. As can be seen in Section 2, the technique is also characterized by a high energy requirement and long processing time. The gases generated in the process, if not captured and reused or controlled, may also have environmental consequences, adding to the carbon footprint. This study, after discussing the common oxides of tin, highlights some emerging beneficiation paths and compares them with conventional ones to predict how viable they could be as alternatives to extracting the metal from cassiterite.

### The Oxides of Tin

The two major oxides of Sn, SnO_2_ and SnO, are well documented, and they are of much interest, probably due to their potential applications in electronics and catalysis. For instance, they have shown great promise for use in sensor materials, transistors, and various forms of conductors (transparent conductors, superconductors, and p-type semi-conductors) [17]. Despite being oxides of the same metal with applications in electronics, they are used for different purposes in these devices due to their intrinsic properties, specifically their valency and atomic coordination. Dai et al. [18] successfully produced SnO diskettes from powders of both oxides; their process followed two mechanisms. The first mechanism is the decomposition of SnO_2_ to gaseous SnO, followed by re-oxidation to SnO_2_. Thus, notwithstanding the high stability of SnO_2_, the decomposition is thermodynamically supported at high temperatures (Equation (1)) [18]. The re-oxidation occurs during the cooling of the gaseous product (Equations (2)–(4)), which has a high thermodynamic feasibility compared to the decomposition in Equation (1), confirming the stability of the +4 state of the oxide [18].

SnO_2(*s*)_ → SnO_(*g*)_ + ½O_2(*g*)_ (1)

SnO_(*g*)_ + ½ O_2(*g*)_ → SnO_2(*s*)_ (2)

SnO_(*g*)_ → ½SnO_2(*s*)_ + ½Sn_(*l*)_ (3)

Sn_(*l*)_ + O_2_ → SnO_2(*s*)_ (4)

The second mechanism is a two-step process for the solid–solid decomposition of SnO to SnO_2_ (Equations (5) and (6)), which occurs simultaneously with the oxidation of Sn_(l)_ to SnO_2_ (Equation (7)). Intermediary tin oxide products (Sn_2_O_3_, Sn_3_O_4_, Sn_4_O_5_, and Sn_5_O_6_) which are mixtures of the +4 and +2 states of the metal are said to occur during this decomposition; however, only Sn_3_O_4_ [(Sn^2+^)_2_(Sn^4+^)O_4_] is known to be stable from a thermodynamic point of view [17,18].

4SnO_(s)_ → Sn_3_O_4(s)_ + Sn_(l)_ (5)

Sn_3_O_4_ → 2SnO_2(s)_ + Sn_(l)_ (6)

Sn_(l)_ + O_2_ → SnO_2(s)_ (7)

The second mechanism is said to proceed in an oxygen-rich atmosphere and usually begins at around 370 °C. In Dai et al.’s study, after 500 and 700 °C treatment for two-and-a-half hours, Sn_(l)_ and Sn_3_O_4_ were not observed [18].

## 2. Conventional Processing of Cassiterite

Conventionally, processing cassiterite requires a two-stage carbothermic reduction process to produce metallic tin through the smelting of the concentrate in the presence of a flux. The first stage (operated at a comparatively lower temperature) simultaneously reduces and smelts the stannic concentrate to produce crude metal tin and a slag. The slag at this stage contains a significant amount of the metal to be discarded; as such, it goes through a second smelting stage, where it is recovered. The slag is essentially a mixture of iron and tin oxide (FeO-SnO) [2]. It may also contain a significant amount of silica and alumina, as well as some critical and strategic metals, such as niobium, tantalum, and tungsten. The smelter conditions, nonetheless, are controlled so that high efficiency and a slag with a suitable FeO composition (30 to 40 wt%) is achieved in the second-stage smelting process [19]. The FeO-to-SnO ratio of the slag is of special significance for achieving a recyclable hardhead with the iron-to-tin ratio required for achieving the ideal flux conditions in the primary smelting process. Generally, a high FeO-to-SnO ratio in both primary and secondary slags is needed to achieve the ideal hardhead for recirculation. A typical slag has the following composition: SnO (3–25%), FeO (10–40%), CaO (5–30%), SiO_2_ (20–40%), and Al_2_O_3_ (up to 10%) [13].

Temperatures up to 1300 °C have been employed at this stage [2,15]. A thermodynamic study by Moosavi-Khoonsari and Mostaghel [2] suggested 1200 °C and a reductant whose quantity equates to a logarithmic oxygen partial pressure (*log*$P_{O_{2}}$) of −12.1 atm as threshold conditions for first-stage smelting if no flux is added. At these conditions, the resulting slag will be composed of about 11% and 33% Sn and Fe, respectively, whilst facilitating Sn and Fe contents of 98 and 2%, respectively, in the crude metal. Increasing the temperature above the threshold (1200 °C) was found to be deleterious, decreasing the Sn recovery through volatilization and entrainment into the slag. Iron which ends up in the crude metal floats; hence, by continuous partial melting followed by crystallization, residual iron can be separated from the metal by skimming. The metal tin obtained is then refined to increase the purity.

The corrosive Sn-rich slag produced (contains 10 to 25% Sn) from the primary smelter moves to the second stage, where harsher reducing conditions (higher temperature and greater reductant and flux amounts) are needed. The slag characteristics and reducing conditions at this stage demand corrosion-resistant equipment for efficient smelting [2,15]. This stage, which is generally carried out at around 1400 °C, produces two products: an iron–tin alloy (FeSn_2_ and/or FeSn), otherwise called a hardhead, and a secondary slag which contains a small quantity of Sn (1 to 2%) and other critical metals [2,15]. It is recommended that, at this operating temperature (1400 °C), a higher reducing condition equivalent to *log*$P_{O_{2}}$ = −11.3 atm is applied to achieve high efficiency [2]. Unlike the first stage, where direct fluxing may not be necessary, appreciable doses of flux are advised in the second stage to reduce slag viscosity, liquidus temperature, and, hence, metal losses. Flux, in the first stage, is achieved through the recycling of the hardhead from the second stage. Gaseous emissions from the system are also crucial and should be closely monitored to enhance efficiency. For instance, the system’s carbon monoxide-to-carbon dioxide ratio (resulting from the Boudouard reaction: C_(s)_ + CO_2(g)_ → 2CO_(g)_) is key in determining the system’s degree of reduction. This is a measure of the actual reduction potential rather than the quantity of reducing agent added. The threshold value of this parameter, if exceeded, may reduce recovery up to 20% through volatilization [2]. The off-gas, especially that from the first stage, therefore needs to be trapped and the fume dust recycled to avoid tin loss through volatilization.

**Figure 1:**
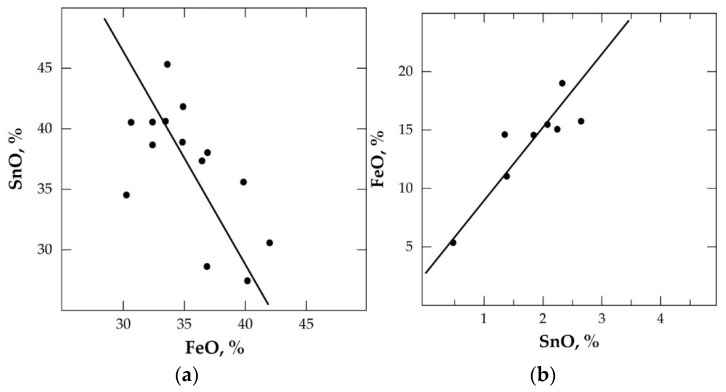
Distribution of SnO and FeO in (**a**) primary slag and (**b**) secondary slag during reductive smelting of cassiterite concentrate.

The hardhead achieved is recycled to the first-stage smelting, whilst the secondary slag is discarded and/or processed to recover other critical metals. Figure 1a,b show the SnO-FeO distribution in the primary and secondary slag, respectively [19].

The distribution is governed by the Fe-Sn metal/slag equilibrium relationship (Equation (8)):

FeO_(slag)_ + Sn_(metal)_ ↔ SnO_(slag)_ + Fe_(metal)_ (8)

**Figure 2:**
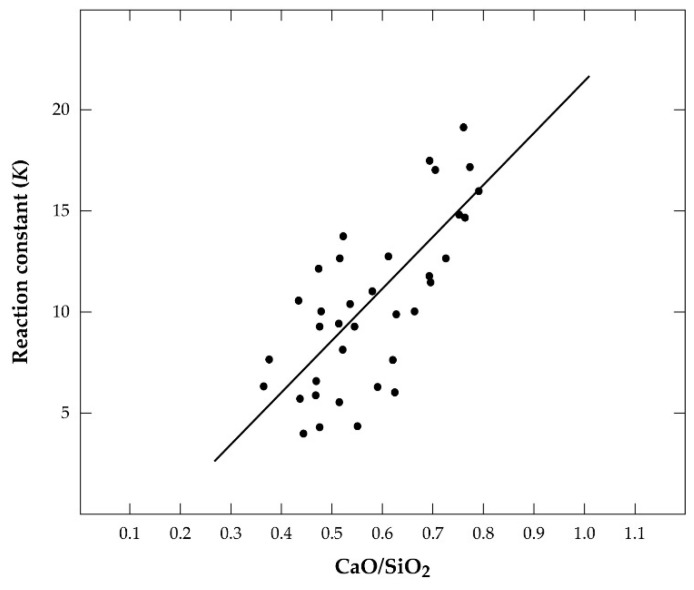
Variation in equilibrium constant, *K* (generated from production data), with CaO-SiO_2_ ratio in the secondary smelter during conventional cassiterite processing.

From the equilibrium constant *K*eq = $\frac{a_{SnO}a_{Fe}}{a_{FeO}a_{Sn}}$, it can be concluded that a direct relation exists between Sn and Fe recovery and either the crude metal or the hardhead. Thus, to avoid the significant dissolution of iron into the crude metal or hardhead, some Sn must also be sacrificed and afforded to the respective slags. This assertion has been confirmed by a further revelation that the phenomenon is more prominent in the later-stage smelting than the former, with the CaO-SiO_2_ ratio in the secondary slag affecting the activity of both FeO and SnO and, hence, the equilibrium constant [2,19]. From production data and linear regression analysis, Equation (9) was developed to relate the equilibrium constant, *K*, CaO-SiO_2_ ratio, and results presented in Figure 2 [19].

*K* = 25.7(CaO/SiO_2_) − 4.34 (9)

**Figure 3:**
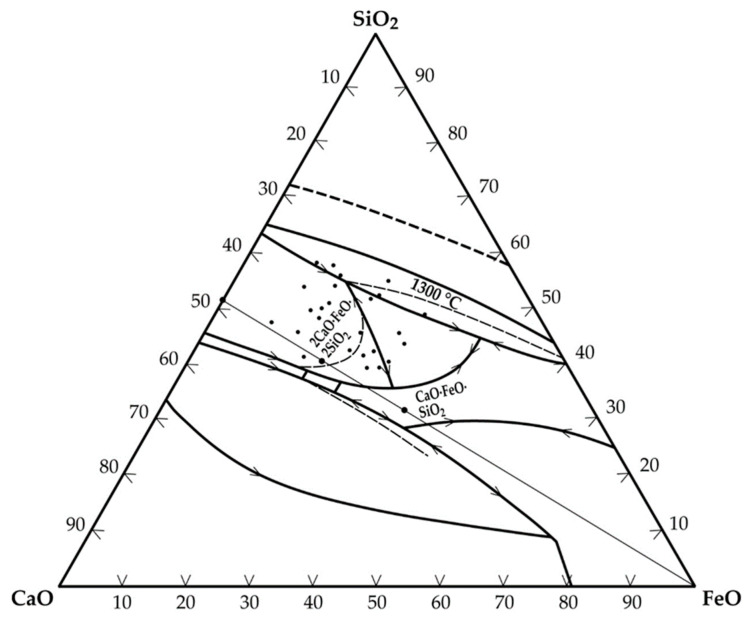
CaO-FeO-SiO_2_ ternary phase diagram indicating slag composition in the secondary smelter during conventional processing and tin recovery from cassiterite.

The correlation coefficient (0.68) obtained for the plot (Figure 2) was, however, low. Thus, in addition to the ratio of iron to tin in the hardhead being a determinant for the FeO-SnO ratio in the slag, the CaO-SiO_2_ ratio also contributes significantly to this phenomenon. Figure 3 is a CaO-FeO-SiO_2_ ternary phase diagram [19] showing the region of typical slag composition in the secondary smelter at a 1300 °C liquidus line.

Fuming by chlorination and sulphidization has been applied as a measure to recover residual tin in hardheads. While chlorination tends to be a corrosive approach, sulphidization has been found to be very efficient for recovering and reducing the tin content in the secondary slag to the bare minimum. To do this, materials with a significantly high sulphur content, such as zinc sulphide, calcium sulphide, iron sulphide, or pyrite (FeS_2_, the commonly used sulphide), are added to the liquid slag [15,20]. The sulphur component reacts with the tin in the slag in a first-order reaction to produce tin sulphide fumes which undergo combustion. The tin oxide formed is cooled and recycled to the first-stage smelter. If, for instance, pyrite is used as the sulphur source, the fuming process needed to yield tin oxide follows Equations (10) and (11).

FeS_2_ + SnO → SnS_2_ + FeO (10)

SnS_2_ + 3O_2_ → SnO_2_ + 2SO_2_ (11)

Different types of furnaces may be used for the fuming, and it is essential that the charge achieves a specific composition regarding the quantity of sulphur-bearing source needed to enhance efficiency. Generally, 70 to 90% tin recovery is possible if 5 to 10% pyrite is used, as was found in [13]. The sulphur source, nonetheless, was found to have a indifferent effect regarding the metal recoveries. The tin fuming modification, if attached to the processing plant, improves recovery; however, it presents some environmental issues through pollution with SO_2_ and heavy metals (which may be present in pyrite), which needs attention.

## 3. Emerging Processes for Extracting Tin from Cassiterite

Cassiterite is not easily amenable to leaching, which makes its processing to recover tin through hydrometallurgical means difficult. It is rare to find a hydrometallurgical approach for recovering the metal from the mineral. It is therefore dominated mostly by high-temperature processes which basically reduce stannic oxide to either the metallic form, as mentioned earlier, or to the stannous form. Other unconventional methods (pyro- or hydrometallurgical, or a combination of both) have been proposed to extract the metal from cassiterite [20].

### 3.1. Sulphuric Acid Leaching of SnO

Metallic properties are more prominent in Sn(II) than Sn(IV), with the divalent states of Sn and Pb being known as the only possible cations in aqueous solution among the group IV elements [20,21]. Therefore, the pyrometallurgical reduction of the stannic to the stannous form prior to leaching may suggest a pyro-hydrometallurgical extraction path. The stannous form is, however, unstable, as it disproportionates at higher temperatures (Equation (12)). To achieve stability, it forms either gaseous stannous, which is tapped out (Equation (13)), or glass when it reacts with silicates in the concentrate, where it is stabilized by the silicate framework.

2SnO → Sn_(l)_ + SnO_2_ (12)

SnO_2_ + Sn_(1)_ → 2SnO_(g)_ (13)

The silicate-bound stannous matrix (glass), if produced, can be processed to recover the metal through crystallization by cooling or seeding and leaching with acids. H_2_SO_4_ leaching of this material (glass) was found to be promising at the optimal conditions of 9 molL^−1^, 60 min, and 70 °C [20]. To obtain a suitable glass composition for this method, it is recommended that (1) a reducing environment rather than a solid reducing agent is employed for the formation of the stannous form in order to prevent a total reduction to metallic tin, which will inhibit leaching, and (2) the feed (concentrate) be appreciably heated to melt (preferably above 1250 °C) before quenching. The glass composition is found to have a significant effect on dissolution, meaning that a very high silica concentration is a disadvantage. In cases where the glass has substantial and unwanted silica, an improvement can be made through the addition of additives such as CaO. That notwithstanding, a minimum of 20% free silica is deemed necessary to enhance the rigidity of the silicate framework. Glass composed of 12.7% Sn, 16.4% Fe, and 33.8% SiO_2_ has been shown to be ideal for leaching, whilst time and temperature have shown a minimal effect on the metal dissolution [20]. The basic challenge of the acid leaching of SnO approach is the non-selectivity of the technique to other metals. That is, metals such as iron and aluminium may be leached alongside tin, which will affect downstream processing.

### 3.2. Cooling Crystallization of SnO

Instead of the hydrometallurgical processing (sulphuric acid leaching) of glass to extract the metal, controlled cooling can be applied instead. This may lead to the segregation of the silicate component with a high iron content leaving behind a tin-rich glassy phase which can be physically separated. Similarly, a seed such as soda or flourite may be added to induce the selective segregation of the tin-rich phase from the silicate component. Conditions including temperatures of around 1000 °C and 120 min of treatment are known to produce a tin-rich phase which can be separated from the silicate gangue but also yield hercynite, which can cause separation challenges [20]. It is suspected that an extended residence time and some additives (such as CaO) can improve the crystallization and separation of tin. A downside of this approach is the high thermal requirement for glass formation. Also, the crystallization and separation of the metal from gangue is very sensitive to the feed composition, which makes realizing industrial applications complicated and not economically viable.

### 3.3. Sulphide Leaching

The high-pressure dissolution of cassiterite with Na_2_S under H_2_S has been proposed for extracting the metal from cassiterite. The sulphur component in some base metal sulphides is found to aid the dissolution of their respective metals, which also suggests that there is another extractive pathway for the mineral. For instance, the dissolution of antimonite (Sb_2_S_3_) and tin sulphide to produce sulphide complexes of the metals has been documented [20,22,23]. The sulphide leaching process required for tin extraction follows Equations (14) and (15), where SnS_2_, after being formed, can be leached using a sulphide lixiviant to give the thiostannate complex (Equation (16)).

½SnO_2_ + H_2_S^(g)^ → ½SnS_2_ + H_2_O (14)

SnO_2_ + CS_2_^(g)^ → SnS_2_ + CO_2_ (15)

SnS_2_ + S_2_^2−^ → SnS_3_^2−^ (16)

All the above reactions (Equations (14)–(16)), from a thermodynamic point of view, are possible even at room temperature, which adds to the feasibility of extracting the metal from the mineral by this approach. In 1971, Nixon [20] experimentally investigated this using three sulphides (Na_2_S·9H_2_O, CaS, and NaHS.H_2_O) on three different grades of simulated cassiterite concentrates (assaying 70, 60, and 18% Sn). The leaching was successful, and a significant amount of the metal was detected in solution, especially when using the high-grade concentrates and Na_2_S·9H_2_O. Leaching of the low-grade concentrate was, however, found not promising, except in cases with an excess of the reagent. CaS and NaHS·H_2_O performed poorly as lixiviants, and their poor performances were attributed to their low solubility and insufficient water of crystallization to aid hydrolysis, respectively. Partially reducing the concentrate prior to the sulphide leaching was found to be very effective, to the point where almost all the reduced portion was dissolved. For the higher kinetics of the entire study, elevated temperatures coupled with high sulphide-to-tin ratios are advised. They recommended this process for high-grade tin concentrates rather than lean grades with temperatures around 300 to 400 °C, and high pressure values are a requirement for its success. An improved recovery close to 98% was achieved when the process was adjusted by adding 50 g/L caustic soda during a 60 min leaching with 200 g/L of Na_2_S·9H_2_O [20]. This process has the ability to eliminate the challenges posed by iron and aluminium, but the non-selectivity of the reagent to silica presents serious challenges during downstream recovery of the metal.

### 3.4. Alkaline Leaching

Some alkali metal hydroxides may leach out tin from the stannic and stannous oxide, as shown by Equations (17) and (18), respectively:

SnO_2_ + MOH → M_2_SnO_3_ + H_2_O (17)

SnO + 2MOH + ½O_2_ → M_2_SnO_3_ + H_2_O (18)

where M is an alkali metal.

It is observed that whilst there is a straightforward dissolution of stannic oxide with the alkali, the stannous oxide form requires an oxidizing environment, as indicated in Ref. [24]. Synergic leaching of the metal may be achieved when this technique is coupled with the sulphide leaching discussed in Section 3.3 above. Pommier and Escalera [25] employed the synergic effect of NaOH and Na_2_S·9H_2_O to leach the metal contained in volatized dust of sulphidic tin-bearing mineral. The mechanism follows Equations (19) and (20) and Equations (21) and (22), where no oxidizing and oxidizing conditions are required for SnO_2_ and SnO, respectively. The SnS formed may also be leached using their respective lixiviation systems in an oxidizing environment (Equations (23) and (24)). Full dissolution of the metal as Na_2_SnO_3_ was achieved at 90 °C treatment for 8 h with continuous agitation.

SnO_2_ + NaOH → Na_2_SnO_3_ + H_2_O (19)

SnO_2_ + 3Na_2_S + H_2_O → Na_2_SnS_3_ + 4NaOH (20)

SnO + 2NaOH + ½O_2_ → Na_2_SnO_3_ + H_2_O (21)

SnO + 3Na_2_S + ⅕O_2_ + 2H_2_O → Na_2_SnS_3_ + 4NaOH (22)

3SnS + 6NaOH + ^3^/₂O_2_ → Na_2_SnS_3_ + 2NaSnO_3_ + 3H_2_O (23)

SnS + 2Na_2_S + ½O_2_ + H_2_O → Na_2_SnS_3_ + 2NaOH (24)

Since the product of the fuming process is SnS, it can suitably be coupled with this process, as the SnS produced from fuming will serve as the feed for this process.

### 3.5. Dry Chlorination

Dry chlorination of cassiterite concentrate with an appropriate chlorination agent may lead to the formation of volatile stannic chloride, as indicated in Equation (25). This technique is less popular for processing cassiterite, though it is especially suitable in cases where the grade of the metal is low [26,27,28,29]. According to a thermodynamic study [30], the presence of a reducing agent facilitates the process, enabling the occurrence of the forward reaction at lower temperatures compared to cases without a reductant. For instance, using chlorine gas, the forward reaction is not likely to occur even at high temperatures (up to 1000 °C), whilst carbochlorination with carbon monoxide and chlorine gas (Equation (26)) achieves reaction feasibility at lower temperatures, even below 100 °C.

½SnO_2_ + Cl_2(g)_ = ½SnCl_4(g)_ + ½O_2(g)_ (25)

½SnO_2_ + Cl_2(g)_ + CO_(g)_ = ½SnCl_4(g)_ + CO_2(g)_ (26)

This approach was confirmed in [19] using a mixture of calcium chloride and coal. The presence of silica in the concentrate was indicated to be essential for the process, forming wollastonite slag, which favours the reaction. The mechanism of this process follows Equations (27) and (28), where the CO produced through Boudoaurd’s reaction reduces the stannic form to the stannous form. The produced stannous-form CO then reacts with calcium chloride and silica, forming stannous chloride and wollastonite slag, as shown in Equation (29). At 900 °C treatment for 180 min, almost all the metal content of the concentrate volatized as stannous chloride.

C + CO_2_ = 2CO (27)

SnO_2_ + CO = SnO + CO_2_ (28)

SnO + CaCl_2_ + SiO_2_ = SnCl_2_ + CaSiO_3_ (29)

## 4. Thermodynamic Insight into Hydrometallurgical Extraction of Tin

Pourbaix diagrams (Eh-pH diagrams) are essential tools used in hydrometallurgy to navigate the leaching conditions of a system. They give an idea of the suitable pH and electrochemical potential (Eh) windows required to achieve the ions of the metal in solution. Metals oxides (such as SnO_2_ or SnO) in aqueous solution interact with water molecules through their O-H bonds, leading to their rapture to form an acidic or basic solution and a new species of the metal. The bond interaction rapture (called hydrolysis) continues, successively leading to the formation of several new species of the metal. In a Sn-H_2_O system, the process can be described by Equations (30)–(34) for SnO_2_ and Equations (35)–(38) for SnO. The resulting products, in order of decreasing acidity, are Sn^4+^, Sn(OH)^3+^, Sn(OH)_4_, Sn(OH)_5_^−^ and SnO_3_^2−^ (for Sn(IV)) and Sn^2+^, Sn(OH)^+^, Sn(OH)_2_, and Sn(OH)_3_^−^ (for Sn(II)). It follows that manipulating the leaching conditions outside the stability region of the metal oxide can help achieve the ions of the metal in solution.

SnO_2_ + 4H^+^ ↔ Sn^4+^ + 2H_2_O (30)

SnO_2_ + 3H^+^ ↔ Sn(OH)^3+^ + H_2_O (31)

SnO_2_ + 2H_2_O ↔ Sn(OH)_4_ (32)

SnO_2_ + 3H_2_O ↔ Sn(OH)_5_^−^ + H^+^ (33)

SnO_2_ + H_2_O ↔ SnO_3_^2−^ + 2H^+^ (34)

SnO + 2H^+^ ↔ Sn^2+^ + H_2_O (35)

SnO + H^+^ ↔ Sn(OH)^+^ (36)

SnO + H_2_O ↔ Sn(OH)_2_ (37)

SnO + 2H_2_O ↔ Sn(OH)_3_^−^ + H^+^ (38)

**Figure 4:**
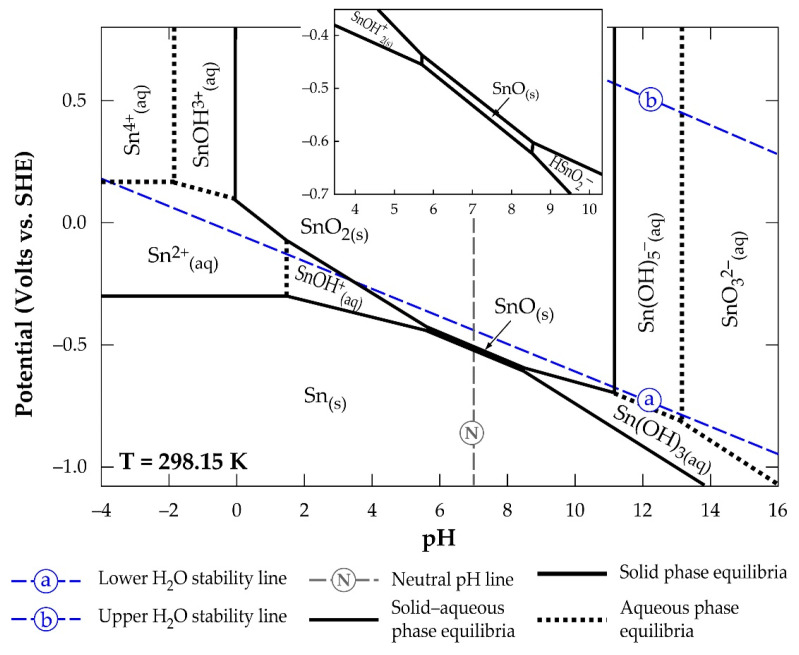
Eh-pH diagram of a Sn-H_2_O system indicating the stability regions of SnO_2_- and SnO-hydrolysed products.

Figure 4 is a Sn-H_2_O Pourbaix diagram constructed by Palazhchenko [31] using literature information gathered at 25 °C.

**Figure 5:**
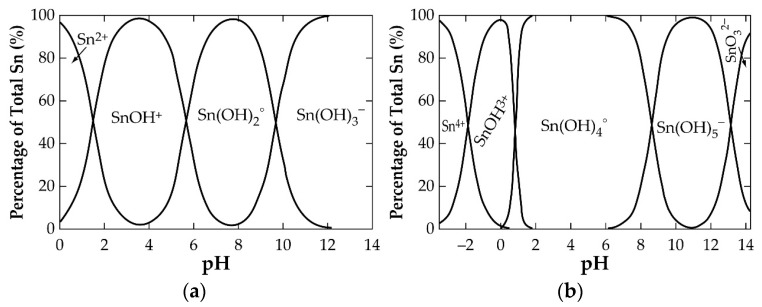
Amounts of (**a**) Sn (II) and (**b**) Sn (IV) speciation products at 25 °C as a function of pH during the hydrolysis of SnO and SnO_2_, respectively.

Palazhchenko’s work shows all the hydrolysis products of the metal, where the final product of the hydrolysis of SnO_2_ is indicated as SnO_3_^2−^, rather than Sn(OH)_6_^2−^, due to the possibility of highly charged metal ions forming oxyanions. The diagram reveals strong acidic conditions and a very small dissolution window for obtaining the Sn(II) and Sn(IV) in solution. One may infer that the use of Sn(IV) dissolution is almost not possible from a look at its stronger acidic requirement and smaller stability region compared to Sn(II). In high-alkaline conditions, the diagram identifies the possibility of achieving the (IV)-state in solution as SnO_3_^2−^ (or Sn(OH)_6_^2−^). It can be concluded that the amphoteric nature of SnO_2_ makes it soluble in highly acidic and alkaline systems. Figure 5 [31] shows the supposed quantities of hydrolysed products of Sn(II) (a) and Sn(IV) (b), as a function of pH at 25 °C, that can be obtained in solution.

Extracted results from a practical work, [31], on the dissolution of Sn(II) and Sn(IV) at 85 °C using powders of Sn(II)- and Sn(IV)-oxides are presented in Table 2.

The Sn(II) dissolution in acidic medium (using HCl and CH_3_COOH) was very weak, to the point that it was below the detection limit (4.0 × 10^−6^ molL^−1^) of the ICP-OES device irrespective of the acid, although the quantity achieved with HCl was slightly higher than that achieved with CH_3_COOH. Augmenting the CH_3_COOH system by purging with nitrogen gas only decreased the redox potential, but no significant effect on the metal’s dissolution was observed. The reaction of this study is modelled after Equation (36), and SnOH^+^ is predicted to be the Sn(II) species produced.

Regarding Sn(IV), the wide stability window of Sn(OH)_4_ (Figure 5b) implies its dominance in solution over its counterparts.

This puts a limitation on the stability windows of other Sn(IV) species, meaning they can actually be measured, except when harsh conditions are applied. In spite of this, a low metal concentration below the detection limit was also observed in the Sn(OH)_4_ stability regions investigated in acidic media, except for HCl at pH = 0.15. CF_3_SO_3_H showed values which were slightly above the detection limit. The high performance of HCl is said to be due to Sn-Cl complex interference at high chloride concentrations. In alkaline media where a NaOH/CH_3_COONa mixture and NaOH were used, the suspected products were Sn(OH)_5_^−^ and SnO_3_^2−^, respectively (Equations (33) and (34)). Whilst there were no literature data for comparing SnO_2_/Sn(OH)_5_^−^ hydrolysis, the solubility constant value of −27.3 ± 0.03 obtained for SnO_2_/SnO_3_^2−^ was indicated to have deviated from literature data.

The consideration of SnO_3_^2−^ as the only species in highly alkaline medium without taking other species into account was given as the cause of this deviation.

The Gibbs free energy change for the acid leaching of the concentrate required to obtain the quadrivalent form of the metal (Equation (39)) confirmed the unlikeliness of the reaction proceeding to the forward direction. It can be suggested that the simultaneous reduction–dissolution of SnO_2_ (Equation (40)) is required to leach the metal in its divalent state from the mineral.

SnO_2_ + 4H^+^ → Sn^4+^ + 2H_2_O (39)

SnO_2_ + *a*H^+^ + *b*R^−^ → Sn^2+^ + *c*H_2_O + *d*R′ (40)

Here, R is the reducing agent, and R′ is its oxidized form.

It is suspected that the acid and reductant type employed (including gases) has a significant effect on the feasibility of the reduction–dissolution reaction of SnO_2_. The use of reducing gases for this technique is said to be thermodynamically feasible; however, pressurized equipment is needed to maintain the required pressure of the system. The effect of some reducing gases has been investigated in this study. In Table 3, (Equations (41)–(46)), reduction–dissolution reactions of some gases and their standard Gibbs free changes have been calculated using HSC Chemistry software^®^ 5.1. Results indicated that, carbon monoxide is the only feasible gas for the leaching reaction, though Ref. [20] indicated feasibility for hydrogen gas for this process. The authors of Ref. [20], suggested a more favourable thermodynamic reaction for hydrogen gas in the presence of HCl. Probably, the Sn^2+^-Cl^−^ complex formation in the presence of HCl could be responsible for this feasibility.

**Table 3:** Reduction–dissolution reactions of some gases and their standard Gibbs free changes.

Leaching Reaction	${\Delta G}_{25{}^{\circ}C}^{o}$ (kJ·mol^−1^)	Equation Number
SnO_2_ + CO_(g)_ + 2H^+^ → Sn^2+^ + H_2_O + CO_2(g)_	−6.006	(41)
SnO_2_ + H_2(g)_ + 2H^+^ → Sn^2+^ + 2H_2_O	14.037	(42)
SnO_2_ + CH_4(g)_ + 8H^+^ → 4Sn^2+^ + 6H_2_O + CO_2(g)_	186.598	(43)
1.5SnO_2_ + NH_3(g)_ + 3H^+^ → 1.5Sn^2+^ + 3H_2_O + 0.5CO_2(g)_	37.466	(44)
SnO_2_ + SO_2(g)_ + 2H^+^ → Sn^2+^ + H_2_O + SO_3(g)_	180.26	(45)
SnO_2_ + H_2_S_(g)_ + 8H^+^ → 4Sn^2+^ + 5H_2_O + SO_3(g)_	429.884	(46)

The Gibbs free energy change, (∆G), is related to its standard conditions, (${\Delta G}^{o}$), in Equation (47):

(47) $$\Delta G={\Delta G}^{o}+RTlnQ$$

where R, T, and Q are the universal gas constant, absolute temperature, and reaction quotient, respectively. Hence, from the general reduction–dissolution reaction (Equation (40)), Equation (47) can be rewritten as follows:

(48) $$\Delta G={\Delta G}^{o}+RTln\frac{\left[Sn2+\right]}{{\left[H^{+}\right]}^{a}{\left[R^{-}\right]}^{b}}$$

It can therefore be inferred from Equation (48) that temperature and the activity of reagents (acid or gas) have an antagonistic effect on the reaction feasibility. Thus, increasing temperature decreases feasibility, and vice versa; increasing the concentration of reagents, on the other hand, increases feasibility, and vice versa.

From a secondary source of the metal (lead-tin solder), HCl was found to exhibit a superior extraction efficiency than H_2_SO_4_ and HNO_3_, achieving 95.97% dissolution of the metal in solution at an estimated activation energy of 117.68 kJ/mol compared to the negligible extraction exhibited with the other acids [32]. Similarly, Moosakazemi et al. [33] achieved an appreciable but lower dissolution (88%) of the metal with HCl when milder conditions (2 molL^−1^ at 75 °C) than the former were used. It can be inferred that using HCl alone as a lixiviant may be suitable for solubilizing the elemental form of the metal, but it becomes complicated in the case of its bearing mineral (cassiterite).

Further investigations revealed that the feasibility of the reduction–dissolution system increases with an increase in the reduction potential of the reductant during leaching. It is therefore possible to suggest that the reductant is the actual leaching agent, whilst the acid helps to stabilize the metal in the bulk solution. Hong [11] in his study used chromium in the presence of some acids to aid leaching of synthetic cassiterite as followed by Equations (49) and (50)


(49) $${\mathrm{SnO}}_{2}+{4H}^{+}+2{\mathrm{Cr}}^{2+}\to {\mathrm{Sn}}^{2+}+2{\mathrm{Cr}}^{3+}+2H_{2}O\to \;{\Delta G}_{25{}^{\circ}C}^{o}=-124.38\;\mathrm{kJ}\cdot {\mathrm{mol}}^{-1}$$

(50) $${\mathrm{SnO}}_{2}+{4H}^{+}+4{\mathrm{Cr}}^{2+}\to \mathrm{Sn}+4{\mathrm{Cr}}^{3+}+2H_{2}O\to \;{\Delta G}_{25{}^{\circ}C}^{o}=-68.921\;\mathrm{kJ}\cdot {\mathrm{mol}}^{-1}$$

The success of this study was confirmed by the thermodynamic feasibility of the reactions. Two pathways were revealed in this study, with one producing Sn^2+^ (Equation (49)) and the other producing Sn in the metallic form (Equation (50)), but the metallic form was the major product. They indicated hydrochloric acid as the most suitable acid for this approach when compared to sulphuric acid and methane sulfonic acid.

Researchers are now, through hydrometallurgical routes, taking advantage of lixiviant’s complexing and stability effect to solubilize and recover tin from cassiterite [34,35,36]. Earlier in 1989, Tongjin [36] revealed the possibility of solubilizing the mineral with HCl and HF in the presence of H_2_. HF was found to exhibit superior leachability than HCl, which was attributed to the high stability of the F–Sn complex compared to Cl–Sn. Omoniyi [35] authored an encouraging report on the dissolution of tin (more than 80%) with HCl from the mineral, whilst Rodliya, on the other hand, indicated only 14% dissolution with the same reagent, although upon the addition of H_2_O_2_, more than 90% efficiency was achieved. The discrepancy in the leaching efficiencies of HCl on the mineral calls for further investigations to ascertain the influence and the leaching mechanism of HCl and other acids on the mineral dissolution. Another study [37], through alkaline smelting of the mineral with a eutectic NaOH/KOH mixture, followed by leaching with distilled or acidified water, claimed to confirm the duality (amphoteric nature) of SnO_2_. They reported an efficient process for the breakdown of the mineral, by which about 97% of the metal content can be extracted, with the extreme pH conditions of the lixiviant favouring the dissolution.

## 5. Recovery and Refinement of Tin

Crude tin obtained after smelting the concentrate may follow pyrometallurgical purification (liquation and boiling, poling) and/or electrorefining. Electrorefining is known to produce purer metal compared to typical pyrometallurgical purification. The metal-laden liquor obtained from hypothetic hydrometallurgical processes (discussed above) or the direct leaching of the metal from waste, on the other hand, can be recovered by electrowinning, precipitation (chemical or hydrolysis), and metal displacement. Recovery by solvent extraction has not, as of now, gained popularity. Impurities of major concern during recovery from solution are mainly iron, arsenic, antimony, copper, and lead. Like any other metal recovery process, it is essential to separate them from solution before the application of the recovery technique.

### 5.1. Conventional/Pyrometallurgical Refining

The crude metal achieved from smelting may be purified or separated from impurities by alternate heating and cooling whilst mixing with air to oxidize the impurities. This results in a mushy dross, which can be skimmed off, leaving a pure metal. The above process, called boiling, can be followed by liquation (usually used to separate impurities with a higher melting point than Sn). To do this, the dross or impure tin containing impurities with higher melting point is heated on a sloping hearth. Tin (having a lower melting point) melts and flows down along the slope to be collected, leaving behind the impurities. In some rare cases, selective distillation may be used to separate impurities by taking advantage of the difference in their boiling point. Here, the impure metal is heated to a very high temperature in a pressurized container. A vacuum may then be introduced to selectively collect the vaporized components based on the boiling points. Elsewhere [34], tossing and poling have been indicated as a pyrometallurgical purification technique. Where needed, electrolytic refining may follow pyrometallurgical purification, which yields very pure metal.

### 5.2. Hydrometallurgical Purification and Recovery of Tin

#### 5.2.1. Chemical Precipitation

Dissolved tin, either in stannous or stannic form, may be recovered from the aqueous solution by the addition of a suitable precipitant. Several reagents, including gases, may be used when different tin precipitates (sulphides, hydroxides, etc.) can be obtained. As discussed earlier, the stannic and stannous metal dissolves at low pH, meaning that, by increasing the pH above 1.0, they can be precipitated out of the solution. Thus, the hydroxides of Sn(II) and Sn(IV) can be precipitated at pH = 1.5 and 0.5, respectively. The pH precipitation windows of these metal hydroxides are almost the same as those of their sulphides, making the recovery of a mixture of both precipitates possible. The difference is that the hydroxide form is gelatinous, whilst the sulphide crystallizes out very well, making the separation of the former difficult compared to the latter. Flotation of the gelatinous tin hydroxide is said to be appropriate for collecting the precipitate in cases where gravity separation becomes challenging.

#### 5.2.2. Hydrolysis Precipitation

In cases where leaching is carried out with sulphuric acid, a mixture of the SnSO_4_ and Sn(SO_4_)_2_ may be precipitated as SnO_2_ by heating at elevated temperatures and under high oxygen pressure. Stannous sulphate has a higher stability than its stannic counterpart; therefore, at around 30 °C, hydrolysis of the latter begins, producing Sn(OH)_4_ and SnO_2_ at temperatures around 60 and 90 °C, respectively. The stannous sulphate, on the other hand, can persist in solution up to 100 °C before it begins to hydrolyse [38]. Hence, a mixture of stannous and stannic sulphate will require temperatures above 90 °C (preferably 110 °C) and oxygen pressure from 5 to 10 p.s.i to precipitate as SnO_2_ from solution. One advantage of this technique is the possible recovery and recycling of sulphuric acid to the system.

#### 5.2.3. Electrowinning

Sn(II) sulphate may also be electrowon rather than precipitated by hydrolysis. The process can be described by Equations (51) and (52) and Equation (53) for the cathode and anode, respectively. Thus, aside from the metal reduction at the cathode, hydrogen gas can be also produced through the competitive reduction of hydrogen ions from the acid at the cathode. Oxygen is the major product at the anode.

2H^+^ + 2e^−^ → H_2_ (51)

Sn^2+^ + 2e^−^ → Sn (52)

4OH ↔ O_2_ + 2H_2_O + 4e^−^ (53)

The concentration of the liquor (stannous sulphate) is said to be very critical to the efficiency and economics of this approach. This is because a liquor resulting from highly concentrated sulphuric acid and Sn(II) forms stable stannous complexes, reducing the quantity of ions which are actually free to be reduced for deposition. Consequently, high potential will be needed to decompose the complexes needed for the reaction to proceed, which, in effect, will increase the processing cost. The increased acid concentration also implies a high hydrogen ion concentration, which may lead to the preferential reduction of hydrogen ions rather than the metal.

Cathode reactions for alkaline electrowinning, according to the lixiviants used by Pommier and Escalera [25], can be represented by Equations (54)–(56).

4H_2_O +4e^−^ → 2H_2_ + 4OH^−^ (54)

SnO_3_^2−^ + 2H_2_ → 2H_2_O + O^2−^ + Sn (55)

SnS_3_^2−^ +2H_2_ → 2H_2_S + S^2−^ + Sn (56)

Their overall process is described by Equations (57) and (58), which, when followed, enable the regeneration and recycling of the lixiviants (NaOH and Na_2_S·9H_2_O) whilst the oxygen is evolved at the anode:

Na_2_SnO_3_ + H_2_O → 2NaOH + O_2_ + Sn (57)

Na_2_SnS_3_ + 2O_2_ → Na_2_S + 2SO_2_ + Sn (58)

Generally, a comparatively low current density (two times less) is needed for electrowinning the metal in acidic media compared to alkaline media.

#### 5.2.4. Metal Displacement

Liquor containing Sn(II) may be recovered from the acidic solution by the displacement of tin by other metals of high reactivity. The divalent state of tin presents a better form for this approach than the quadrivalent, considering that a small quantity of iron is needed to reduce the former compared to the later.

It is required that a dilute acid and a metal of high electrode potential be used. This will prevent a favourable hydrogen discharge reaction, leading to the evolution hydrogen gas rather than the reduction of the metal. Zinc may be a suitable metal for this technique, but its cost means it is not economically viable for the metal’s recovery. Iron, according to Ref. [20], should be an economical metal to use, but the authors of this study highlighted some inefficiencies which make it not suitable. These inefficiencies notwithstanding, Fitzhugh et al. [39] posited that the recovery of the metal with iron is possible. Stannous chloride solution obtained by the chlorination of a tin-bearing material with HCl or a suitable chlorination agent was used for this invention. Factors influencing the recovery included temperature and solution pH, whilst iron particle size and iron purity were found to affect the rate of the reaction. At optimal temperatures between 115 and 150 °C and pH values ranging from 1.4 to 1.6, about 99.5% recovery was achieved [39]. One critical requirement for the success of the iron displacement of tin is the use of a pressurized reactor to trap and maintain the hydrogen partial pressure from the gas that evolves.

## 6. Conclusions

The extractive metallurgy of tin from cassiterite is dominated by the reductive smelting of the mineral in the presence of flux. The major challenges encountered by this method are the difficulty in separating concomitant iron from the crude metal tin without affecting recovery and inefficiencies resulting from equipment design. The actual determinant for the extent of reduction of the mineral is estimated based on the ratio of the carbon monoxide to carbon dioxide which is produced in the system during operation. This study has led us to the conclusion that, aside from the conventional method, all the proposed extractive pathways for the mineral have inherent challenges which do not allow for industrial application, making the reductive smelting route the sole economic process for industry. This notwithstanding, sulphide leaching promises some success, especially for partially reduced concentrates, and alleviates the non-selective challenge posed by sulphuric acid leaching. If glass is to be processed to recover its tin content, leaching with sulphuric acid, as discussed above, is an advisable method.

This study has revealed reductive-dissolution as an encouraging path for solubilizing tin from the mineral. H_2_ has, so far and to the best of our knowledge, been identified as the only reducing gas that can be used for this type of study, but our thermodynamic investigation revealed CO to be very effective for this task. We therefore recommend that attention be given to CO coupled with the already used HCl or HF, as well as other acids, to investigate how they perform in terms of leaching the mineral.

Cassiterite is mostly associated with some critical metals, such as niobium and tantalum, which mostly end up in the slag at the end of processing. The slags are processed differently to recover niobium and tantalum. It is therefore recommended that future studies look into possible ways to recover critical metals, alongside tin, from the mineral in a single process, like the selective chlorination process proposed by the EXCEED Horizon Europe project, the development of which is ongoing, and it can be used to separate and refine critical metals and tin from cassiterite concentrate.

## Figures and Tables

**Table 1:** Composition of cassiterite concentrate.

Oxide	wt%
SnO_2_	75.85
SiO_2_	8.85
TiO_2_	3.54
Fe_2_O_3_	2.75
Al_2_O_3_	1.88
MgO	1.85
Others	5.28

**Table 2:** Logarithm of SnO and SnO_2_ solubility constant measured at 85 °C.

Medium	pH (±0.01)	Log_10_K°	Suspected Equilibrium Reaction
		**SnO**	
HCl	3.23	−3	SnO + H^+^ ↔ SnOH^+^
CH_3_COOH	2.99	−3	
CH_3_COOH + N_2_	2.94	−3	
NaOH	8.98	−13.75 ± 0.08	SnO + 2H_2_O ↔ Sn(OH)_3_^−^ + H^+^
**SnO_2_**			
HCl	0.15	−5.29 ± 0.02	SnO_2_ + 3H^+^ ↔ SnOH^3+^
CF_3_SO_3_H	0.05	−6.8 ± 0.9	
NaOH/CH_3_COONa	10.38	−15.2 ± 0.1	SnO_2_ + 3H_2_O^+^ ↔ Sn(OH)_5_^−^ + H^+^
NaOH	11.20	−27.3 ± 0.03	SnO_2_ + H_2_O^+^ ↔ SnO_3_^2−^ + 2H^+^

## Data Availability

Research data is available upon request.
